# Supplementary material for: From sequence to enzyme mechanism using multi-label machine learning
Source: BMC Bioinformatics. 2014 May 19;15:150. doi: 10.1186/1471-2105-15-150 (PMC4229970; doi:10.1186/1471-2105-15-150)
Supplement: Additional file 2 — Java code of ml2db. Additional file ml2db_code.tar.gz contains the Java source code to run the multi-label machine learning experiments and save the results to database. The code’s Javadoc is included. [file 1471-2105-15-150-S2.zip › additional file 2/ml2db/ml2db/doc/test/ml2db/dataharness/DataTableTest.html]

DataTableTest


JavaScript is disabled on your browser.


- Overview
- Package
- Class
- Use
- Tree
- Deprecated
- Index
- Help

- Prev Class
- Next Class

- Frames
- No Frames

- All Classes

- Summary:
- Nested |
- Field |
- Constr |
- Method

- Detail:
- Field |
- Constr |
- Method


test.ml2db.dataharness

## Class DataTableTest

- java.lang.Object
- - junit.framework.Assert
  - - junit.framework.TestCase
    - - test.ml2db.dataharness.DataTableTest

- All Implemented Interfaces:
  :   junit.framework.Test

  Direct Known Subclasses:
  :   DataTableFourTest, DataTableOneTest, DataTableThreeTest, DataTableTwoTest

  ---

    

  ```
  public class DataTableTest
  extends junit.framework.TestCase
  ```

  Create a table for test instances (and their attributes and classes)

- - ### Field Summary

    Fields

    | Modifier and Type | Field and Description |
    | --- | --- |
    | `static java.lang.String` | `ATTRIBUTE_FIELD` |
    | `static java.lang.String` | `CLASS_FIELD` |
    | `static java.lang.String` | `INSTANCE_FIELD` |
  - ### Constructor Summary

    Constructors

    Constructor and Description

    `DataTableTest()`
  - ### Method Summary

    Methods

    | Modifier and Type | Method and Description |
    | --- | --- |
    | `void` | `createTestTable(java.lang.String tableName)` |
    | `java.lang.String` | `getInsertDataSqlStatement()` |
    | `uk.ac.standrews.utils.main.database.Table` | `getTable()` |
    | `java.lang.String` | `getTableName()` |
    | `uk.ac.standrews.utils.main.database.TableReader` | `getTableReader()` |
    | `void` | `initialise(java.lang.String tableName)` |
    | `void` | `setTableName(java.lang.String tableName)` |
    | `void` | `testInstanceAttributeValueIsInTable(java.lang.String instance, java.lang.String attribute, java.lang.String value)` |
    | `void` | `testTableInstanceAttribute(java.lang.String instance, java.lang.String attribute)` |
    | `void` | `testTableInstanceClass(java.lang.String instance, java.lang.String classLabel)` |

    - ### Methods inherited from class junit.framework.TestCase

      `countTestCases, getName, run, run, runBare, setName, toString`
    - ### Methods inherited from class junit.framework.Assert

      `assertEquals, assertEquals, assertEquals, assertEquals, assertEquals, assertEquals, assertEquals, assertEquals, assertEquals, assertEquals, assertEquals, assertEquals, assertEquals, assertEquals, assertEquals, assertEquals, assertEquals, assertEquals, assertEquals, assertEquals, assertFalse, assertFalse, assertNotNull, assertNotNull, assertNotSame, assertNotSame, assertNull, assertNull, assertSame, assertSame, assertTrue, assertTrue, fail, fail, failNotEquals, failNotSame, failSame, format`
    - ### Methods inherited from class java.lang.Object

      `equals, getClass, hashCode, notify, notifyAll, wait, wait, wait`

- - ### Field Detail


    - #### INSTANCE\_FIELD

      ```
      public static java.lang.String INSTANCE_FIELD
      ```


    - #### ATTRIBUTE\_FIELD

      ```
      public static java.lang.String ATTRIBUTE_FIELD
      ```


    - #### CLASS\_FIELD

      ```
      public static java.lang.String CLASS_FIELD
      ```
  - ### Constructor Detail


    - #### DataTableTest

      ```
      public DataTableTest()
      ```
  - ### Method Detail


    - #### createTestTable

      ```
      public void createTestTable(java.lang.String tableName)
      ```


    - #### getInsertDataSqlStatement

      ```
      public java.lang.String getInsertDataSqlStatement()
      ```


    - #### getTable

      ```
      public uk.ac.standrews.utils.main.database.Table getTable()
      ```


    - #### getTableName

      ```
      public java.lang.String getTableName()
      ```


    - #### getTableReader

      ```
      public uk.ac.standrews.utils.main.database.TableReader getTableReader()
      ```


    - #### initialise

      ```
      public void initialise(java.lang.String tableName)
      ```


    - #### setTableName

      ```
      public void setTableName(java.lang.String tableName)
      ```


    - #### testInstanceAttributeValueIsInTable

      ```
      public void testInstanceAttributeValueIsInTable(java.lang.String instance,
                                             java.lang.String attribute,
                                             java.lang.String value)
      ```


    - #### testTableInstanceAttribute

      ```
      public void testTableInstanceAttribute(java.lang.String instance,
                                    java.lang.String attribute)
      ```


    - #### testTableInstanceClass

      ```
      public void testTableInstanceClass(java.lang.String instance,
                                java.lang.String classLabel)
      ```


- Overview
- Package
- Class
- Use
- Tree
- Deprecated
- Index
- Help

- Prev Class
- Next Class

- Frames
- No Frames

- All Classes

- Summary:
- Nested |
- Field |
- Constr |
- Method

- Detail:
- Field |
- Constr |
- Method
